# Role of chemokines and their receptors in lesional CD8⁺ T cell homing in Indian Post-Kala-Azar Dermal Leishmaniasis

**DOI:** 10.1371/journal.pntd.0014474

**Published:** 2026-07-24

**Authors:** Shriya Saha, Deep Goswami, Mehelana Saha, Bidhan Chakraborty, Soham Saha, Saikat Karuri, Madhurima Roy, Surya Jyati Chaudhuri, Nilay Kanti Das, Uttara Chatterjee, Raghunath Chatterjee, Mitali Chatterjee

**Affiliations:** 1 Department of Pharmacology, Kolkata, India; 2 Multidisciplinary Research Unit, Kolkata, India; 3 Human Genetics Unit, Indian Statistical Institute, Kolkata, India; 4 Department of Microbiology, Sarat Chandra Chattopadhyay Govt. Medical College and Hospital, Uluberia, Howrah, India; 5 Department of Dermatology, College of Medicine and Sagore Datta Hospital, Kolkata, India; 6 Department of Pathology, Institute of Postgraduate Medical Education and Research, Kolkata, India; Fundação Oswaldo Cruz: Fundacao Oswaldo Cruz, BRAZIL

## Abstract

**Background:**

Post-kala-azar dermal leishmaniasis (PKDL), a dermal sequel of visceral leishmaniasis (VL) is considered as a reservoir that facilitates the transmission of VL. Although PKDL lesions demonstrate an overwhelming infiltration of CD8 ⁺ T cells, characterization of lesional CD8 ⁺ T cells, their tissue-homing chemokine receptors and corresponding ligands remains poorly defined, and was the aim of this study.

**Methodology:**

In patients with PKDL, CD8 ⁺ T cells were phenotyped in skin biopsies, in terms of senescence (CD8 ⁺ /CD57⁺), and cytotoxicity (expression of Perforin and Granzyme). The plasma levels of T cell chemoattractants (CCL3/4/5/17, CXCL9/10), and associated cytokines (IFN-γ, IL-5, TNF-α, IL-15) were assessed by a multiplex assay, while their lesional expression was evaluated by bulk RNA sequencing and immunohistochemistry. Furthermore, the expression of circulating chemokine receptors, namely CCR4 (for CCL17/22), CCR5 (for CCL3), and CXCR3 (for CXCL9/10), were assessed by flow cytometry, and at lesional sites using transcriptomic data/immunofluorescence.

**Principal findings:**

As compared to healthy controls, dermal lesions from PKDL patients showed a significant increase in CD8 ⁺ T cells that demonstrated exhaustion/senescence (CD8 ⁺ /CD57⁺), as also lacked Perforin and Granzyme. In circulation and lesional tissues, T cell chemoattractants (CCL 3/4/5/17, CXCL 9/10) and pro-inflammatory cytokines (IFN-γ, IL-5, IL-15 TNF-α) along with their corresponding receptors, CCR4, CCR5 and CXCR3 were elevated.

**Conclusions:**

In dermal lesions of PKDL, an enhanced homing of CD8 ⁺ T cells was achieved via an upregulation of T cell chemoattractants CCL3/4/5/17, CXCL9/10, and their corresponding receptors. Their exhausted/senescent phenotype and loss of cytotoxicity possibly facilitated parasite persistence, highlighting the need for host directed immunotherapeutic approaches aimed at restoring T cell potency.

## Author summary

Post-kala-azar dermal leishmaniasis PKDL is a dermatological complication that develops after apparent cure from kala-azar/visceral leishmaniasis, caused by the parasite *Leishmania donovani*. It is recognized as an important reservoir for ongoing parasite transmission and thereby poses a significant threat to the success of the ongoing kala-azar elimination program. Moreover, the absence of an animal model in PKDL poses a great challenge in elucidating its pathogenesis, thereby restricting studies exclusively to clinical cases. PKDL lesions are characterized by a dense infiltration of CD8 ⁺ T cells, but their functional status and homing mechanism(s) remain poorly delineated. In this study, skin biopsies and plasma from patients with polymorphic and macular PKDL demonstrated an elevated level of T cell attracting chemokines and their corresponding receptors in circulation and skin lesions that facilitated the dermal homing of CD8 ⁺ T cells. This strong chemotactic gradient between dermis and circulation promoted the recruitment of CD8 ⁺ T cells to the lesional sites. However, despite their accumulation, CD8^+^ T cells displayed features of exhaustion and senescence, along with a reduced expression of key cytotoxic molecules, features necessary for parasite killing. Understanding this immune dysregulation is necessary for development of host-directed therapeutic strategies, which can facilitate elimination of this skin neglected tropical disease.

## Introduction

Leishmaniases, caused by protozoan parasites of the genus *Leishmania*, represent a diverse group of neglected tropical diseases with clinical outcomes ranging from self-healing cutaneous forms to the fatal, if untreated, visceral form. Post kala-azar dermal leishmaniasis (PKDL), a dermal sequel of apparently cured cases of Visceral Leishmaniasis (VL) is caused by *Leishmania donovani* and remains the least understood and most challenging manifestation [1,2]. PKDL typically occurs in apparently cured VL patients and presents with polymorphic (nodules, papules and/or macules) or macular (hypopigmented) lesions [3–5]. These lesions harbour parasites in the skin, making patients active reservoirs for transmission. In South Asia, 2.5–20% of apparently cured VL patients develop PKDL, whereas in Sudan the condition appears in 20–50% of cases, often concurrently with VL [3,6].

The development of leishmaniasis hinges on the inability of host microbicidal mechanisms to eliminate parasites. The outcome of *Leishmania* infection is determined by the balance amongst distinct T cell subsets, including cytotoxic T cells and T helper cell populations [7]. CD4 ⁺ T cells producing IFN-γ play a central role by activating infected macrophages to generate microbicidal molecules, nitric oxide and reactive oxygen species that kill intracellular amastigotes, a classical example being cutaneous leishmaniasis [8]. CD8 ⁺ T cells also contribute significantly by recognizing infected cells through MHC I-presented antigens and, upon receiving appropriate co-stimulatory signals, differentiate into cytotoxic effector cells that produce inflammatory cytokines, along with cytolytic molecules, perforin and granzyme, which promote parasite clearance as also contribute towards tissue pathology [9].

In PKDL, the immune response is characterized by a dysregulated, mixed Th1/Th2 profile with a dominant Th2 bias, reflected in elevated levels of IL-4, IL-5, IL-13, IL-10, and TGF-β coupled with an increased frequency of circulating CD8 ⁺ IL-10 ⁺ T cells [10–13]. In the context of PKDL, studies have focused on characterization of the immune landscape and the inflammatory milieu [13–15], wherein an increased proportion of lesional CD8^+^ T cells, increased levels of circulatory Th1/Th2 cytokines and elevated levels of chemokine receptor CCR4 within the CD8^+^ T cell population was identified [16]. However, in PKDL, in the absence of an animal model, a detailed characterization of lesional CD8 ⁺ T cells, associated homing chemokine receptors and their association with corresponding ligands, remains poorly delineated. Accordingly, this study aimed to assess in patients with PKDL the status of molecular markers related to the migration of CD8^+^ T cells to lesional sites.

## Results

### Study population

The study population (n = 24) included a comparable proportion of patients with PKDL having polymorphic (n = 13) or macular (n = 11) lesions, so as to represent the current clinical scenario reported in West Bengal [3,4]. All cases were naïve and had not received any treatment at the time of collection of skin biopsies and/or blood. All cases reported a prior history of VL, and the median lag period, i.e., gap between appearance of PKDL lesions and completion of treatment for VL, was slightly higher in the polymorphic form *vis-à-vis* the macular form (Table 1). Majority of the cases were sourced following active surveillance (n = 21/24, 87.5%), and a male preponderance was evident in the polymorphic form (Table 1). In terms of treatment received during VL, 13/24 (54.16%, polymorphic, P: macular, M = 8:5) received sodium antimony gluconate (SAG), whereas a comparable proportion received Miltefosine (n = 6/24, 25% P:M = 2:4) or Liposomal Amphotericin B (LAmB, n = 5/24, 20.8% P:M = 3:2). For peripheral blood, healthy volunteers (n = 9) were recruited from non-endemic areas, and were confirmed as negative for anti-leishmanial antibodies. (Table 1). The parasite load as quantified by qPCR was 1.74 fold higher in the polymorphic cases as compared to the macular form [17] (Table 1).

**Table 1:** Study population.

Characteristics	Patients with PKDL		Healthy controls (n = 17)		
	Polymorphic (n = 13)	Macular (n = 11)	Skin biopsy Preputial skin (n = 6)	Skin biopsy Normal skin (n = 2)	Peripheral blood (n = 9)
**Age (years)** ^**a**^	30.00 (27.00-47.00)	39.00 (27.00-59.50)	3.00 (1.50-4.5)	31.5^b^	32.00 (27.0-35.0)
**Gender (M:F)**	12:1	5:6	All males	1:1	2:7
**Disease duration (years)** ^**a**^	2.00 (1.00-5.00)	2.00 (1.00-4.75)	NA	NA	NA
**Interval between VL and** **PKDL (years)** ^**a**^	3.00 (0.25-7.0)	2.00 (1.00-2.75)	NA	NA	NA
**Parasite load (parasites/ µg µg genomic DNA)** ^**a**^	9413.00 (625.00-3435119.00)	5397.00 (983.50-20089.00)	NA	NA	NA

F, female; M, male; NA, not applicable; PKDL, post Kala-azar Dermal Leishmaniasis; VL, visceral leishmaniasis. ^a^values are expressed as median (IQR). ^b^value is expressed as mean.

### Status of cellular infiltrate

**Fig 1:**
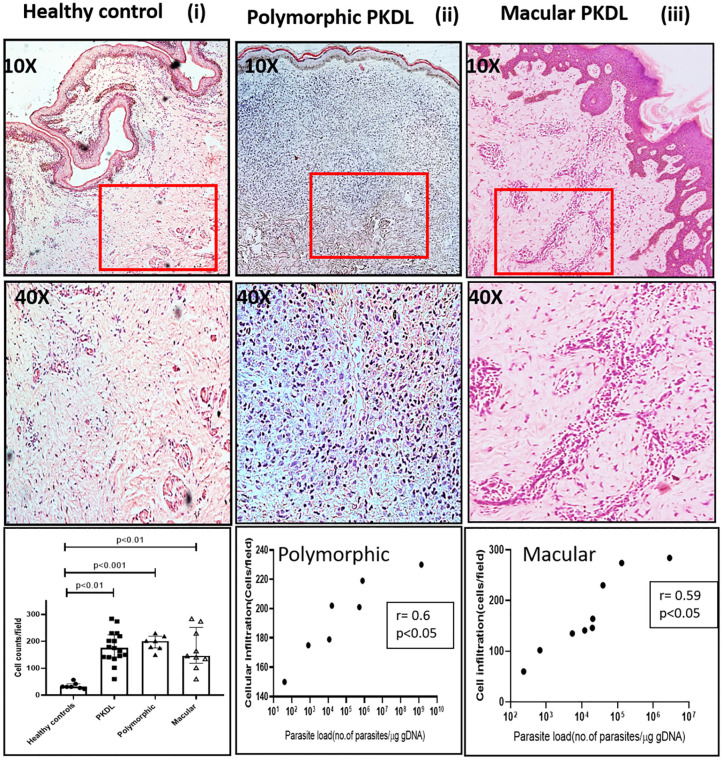
Immune cell enrichment is a feature of PKDL. **i-iii:** Representative immunohistochemical profiles of the cellular infiltrate in dermal biopsies of a healthy control **(i)**, patient with polymorphic **(ii)**, or macular PKDL **(iii)**, (10x and 40x magnification), along with their bar scatter plots indicating the cellular infiltrate in healthy controls (●, n = 6) and patients with PKDL (■, n = 16), either polymorphic (▲, n = 7) or macular, (△, n = 9) forms; each horizontal bar represents the median (IQR). Correlation of cell infiltration (counts/field) with parasite load (no. of parasites/µg genomic DNA) of patients with polymorphic PKDL **(ii)** and macular PKDL **(iii)**.

The cellular infiltrate was diffusely distributed in polymorphic PKDL and was mainly confined to the dermis. Similarly, in the macular form, the infiltrate was located in the dermis, but more clustered, mainly perivascular and periappendegeal. In comparison to healthy controls, the degree of cellular infiltration in PKDL was 4.7 fold higher being [148.0 (112.0-201.0) vs. [31.5 (26.0-43.2) cells/field (Fig 1i), evident in polymorphic and macular forms being [201.5(176.0-224.4) and [144.0(126.30-241.0) cells/field, respectively (**Fig 1**ii, 1iii). For healthy controls, preputial skin was used which has similar skin histology with skin of adults [5,18]. The cellular infiltrate in the polymorphic and macular forms significantly correlated with the parasite load, being r = 0.6 and r = 0.59 respectively (**Fig 1**ii, 1iii).

### Status of CD8^+^ T cells in patients with PKDL

**Fig 2:**
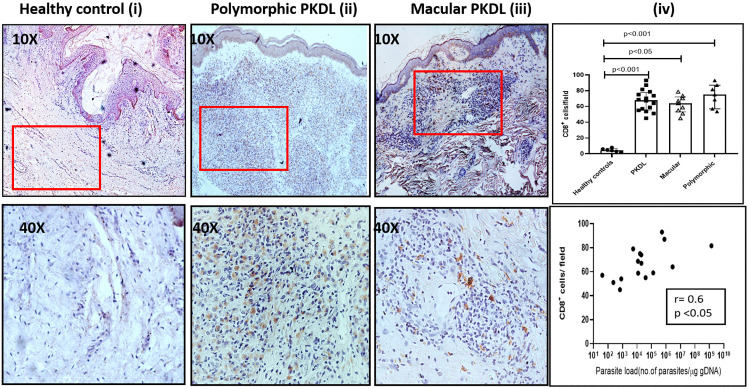
Increased CD8^+^ T cell infiltration in PKDL. i-iii: Representative immunohistochemical profiles of CD8^+^ T cell infiltration from dermal biopsies of a healthy control (i), patient with polymorphic (ii), or macular PKDL (iii), 10x and 40x magnification along with bar scatter graphs indicating the infiltration of CD8^+^ T cells in healthy controls (●, n = 6), and patients with PKDL (■, n = 16), either polymorphic (▲, n = 7) or macular (△, n = 9) forms; each horizontal bar represents the median (IQR). Correlation of CD8^+^ T cells (counts/field) with parasite load (no. of parasites/µg genomic DNA) of patients with PKDL.

In dermal lesions of patients with PKDL, an overwhelming infiltration of CD8^+^ T cells lesions has been reported [5,13,16] and was endorsed in this study, being [64.0(53.5-75.8) cells/field] vs. [3.5(2.2–6.4) cells/field in healthy controls (**Fig 2**i). On an individual basis, density of the infiltrate in polymorphic and macular PKDL was comparable being [71.0(54.0-81.0) and, [62.0(56.0-71.0) cells/field respectively (**Fig 2**ii, 2iii). In both forms, the CD8^+^ T cells were primarily in the dermis, and showed a strong correlation with the parasite load being r = 0.6 (**Fig 2**). As the number of CD8^+^ T cells was comparable between the two forms, subsequent analyses were done considering PKDL as a single entity.

### Overexpression of genes encoding for T cell homing receptors and its ligands in PKDL lesions

**Fig 3:**
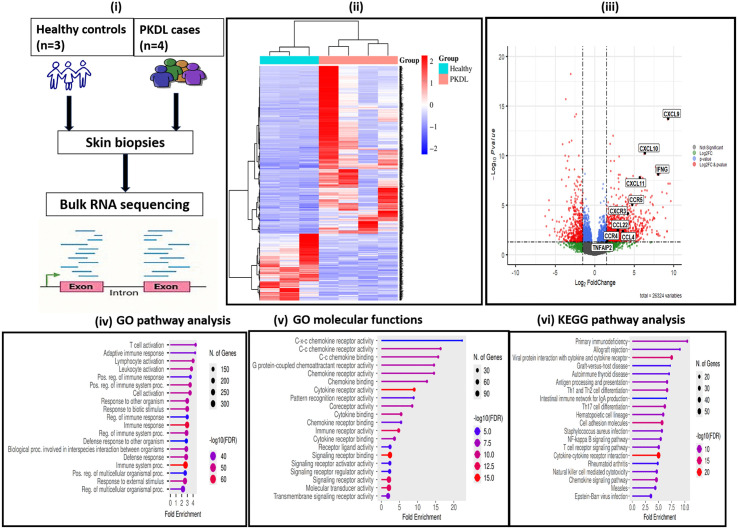
Enhanced dermal expression of T cells associated chemoattractants and receptors in patients with PKDL. **i:** Study design for the bulk RNA-sequencing from dermal lesions of patients with PKDL (n = 4) and healthy controls (n = 3). **ii, iii:** Heatmap **(ii)** and volcano plot **(iii)** of differentially expressed genes (|log₂ fold change| ≥ 1.5 and p-value ≤ 0.05) in skin biopsies from patients with PKDL relative to healthy controls. **(iv-vi)** Pathway analysis of DEGs using databases like Gene Ontology Biological Processes (GO BP**) (iv),** Gene Ontology Molecular Processes (GO MF) **(v)**, and the Kyoto Encyclopedia of Genes and Genomes (KEGG) **(vi).**

In order to characterize what drives the migration of CD8^+^ T cells to lesions, total RNA-sequencing analysis was performed on skin biopsies sourced from PKDL lesions and healthy controls (Fig 3i). A total of 1714 differentially expressed genes (DEGs) were identified between the lesional skin of PKDL cases vs. healthy individuals, 1227 being upregulated and 487 downregulated. This was based on the filtering thresholds of |log₂ fold change| ≥ 1.5 and p-value ≤ 0.05 **(Fig 3**ii, 3iii), as evident in the heat map and volcano plot of the total 1714 DEGs transcriptome data **(Fig 3**ii, 3iii). The volcano plot demonstrated that IFN-γ and several IFN-γ related T cell chemoattractants, along with their corresponding receptors, were significantly upregulated in PKDL as compared to healthy controls **(Fig 3**iii). Gene enrichment analysis was performed on the DEGs and significantly enriched pathways were identified within GO Biological Processes, GO Molecular functions and KEGG. In PKDL, the most significantly enriched terms included cytokine/chemokine receptor binding and their activity, receptor-ligand binding and signalling along with T cell receptor signalling pathways **(Fig 3**iv-3vi).

Cytokines and chemokines play a crucial role in initiating inflammatory responses by attracting CD8^+^T cells from circulation to the lesional sites. A list of cytokines/chemokines (p value ≤ 0.05, |log₂ fold change| ≥ 1.5) associated with T cell homing was created and fold change of expression in terms of log_2_ CPM was evaluated (Table 2). Among the T cell associated cytokines/chemokines studied, *CCL4/17/22, CXCL9/10/11, IL-15, TNF-α and IFN-ϒ* were differentially expressed in PKDL lesions as compared to healthy skin. Accordingly, the expression of corresponding chemokine receptors *CXCR3, CCR4/5, TNFRSF-1B* was determined. The expression of these receptors was elevated in PKDL, as compared to healthy controls, being 11.8-fold for *CXCR3*, 2.2 fold for *CCR4* and 7.3 fold for *CCR5*, 1.5 fold for *TNFRSF-1B* (Table 2).

**Table 2:** Transcriptomic profile of T cells chemoattractants in patients with PKDL.

Name of cytokines/chemokines with corresponding receptors	Fold change (log_2_CPM)
*CCL4 (CCR 5)*	3.3 (7.3)
*CCL22 (CCR4)*	2.3 (2.2)
*CXCL9 (CXCR3)*	11.5(11.8)
*CXCL10 (CXCR3)*	7.9 (11.8)
*CXCL11 (CXCR3)*	12.7 (11.8)
*IL-15 (IL-15Rα)*	2.2 (1.5)
*IFN-ϒ* (IFN- *ϒR)*	28.3 (Nil)
*TNFAIP2 (TNFRSF-1B)*	1.5 (1.5)

The transcriptomic status of T cell related cytokines/chemokines along with their corresponding receptors (in brackets) was assessed in dermal lesions of patients with PKDL and healthy controls as described in materials and methods. Data was assessed in terms of fold change.

### Status of lesional T cells chemoattractants and their receptors in PKDL

**Fig 4:**
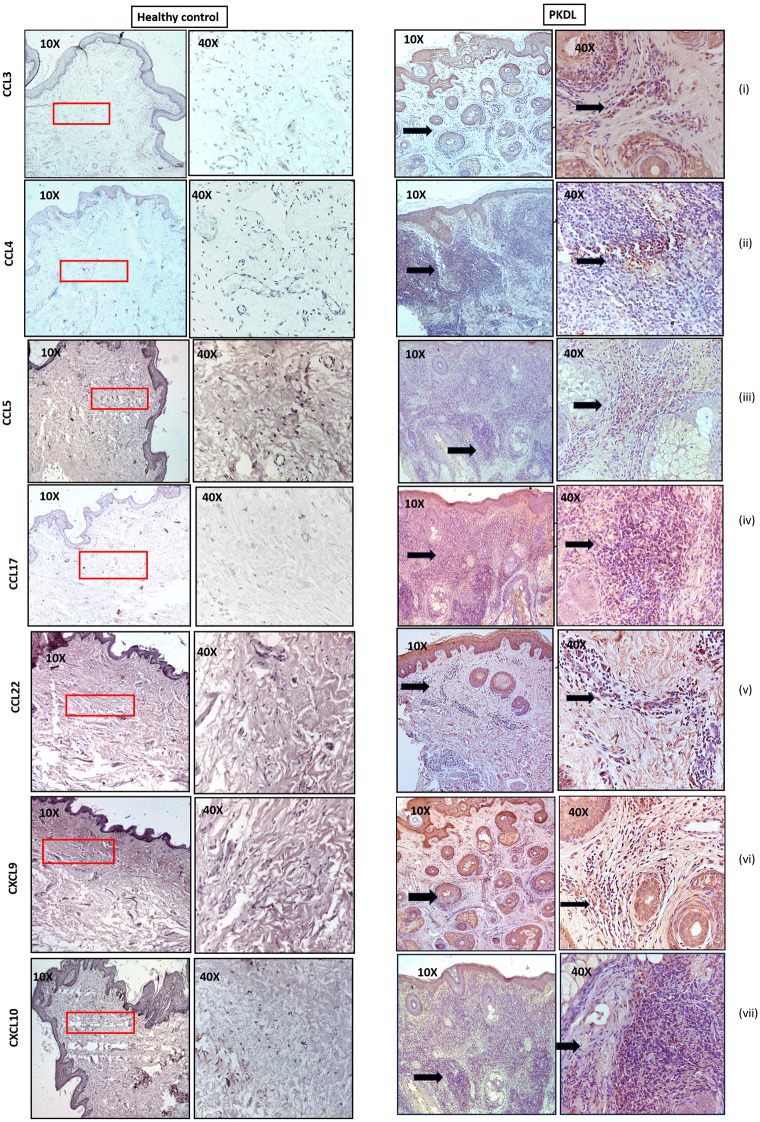
Status of lesional T cells chemo-attractants in PKDL. **i-vii**: Representative immunohistochemical profiles showing the expression of chemoattractants CCL3 **(i)**, CCL4 **(ii)**, CCL5 **(iii)**, CCL17(iv), CCL22 **(v)**, CXCL9 (vi) and CXCL10 (vii) in skin biopsies of a healthy control (n = 6) and patient with PKDL (n = 16, 10X magnification). In PKDL, areas with positive staining were further imaged at 40X magnification. The brown colour indicates a positive stain and is indicated with black arrows.

To substantiate the transcriptomic data, immunohistochemical analysis of dermal lesions was performed. There was a significantly higher proportion of CCL3 **(Fig 4**i), CCL4 **(Fig 4**ii), CCL5 **(Fig 4**iii), CCL17 **(Fig 4**iv), CCL22 **(Fig 4**v), CXCL9 **(Fig 4**vi) and CXCL10 **(Fig 4**vii) positive cells, largely confined to the dermis, *vis-a-vis* their near total absence in healthy controls. As the expression of chemokines was diffuse, individual cells were not counted; hence, a quantitative correlation, if any, between chemokines and the number of CD8^+^ T cells was not attempted.

**Fig 5:**
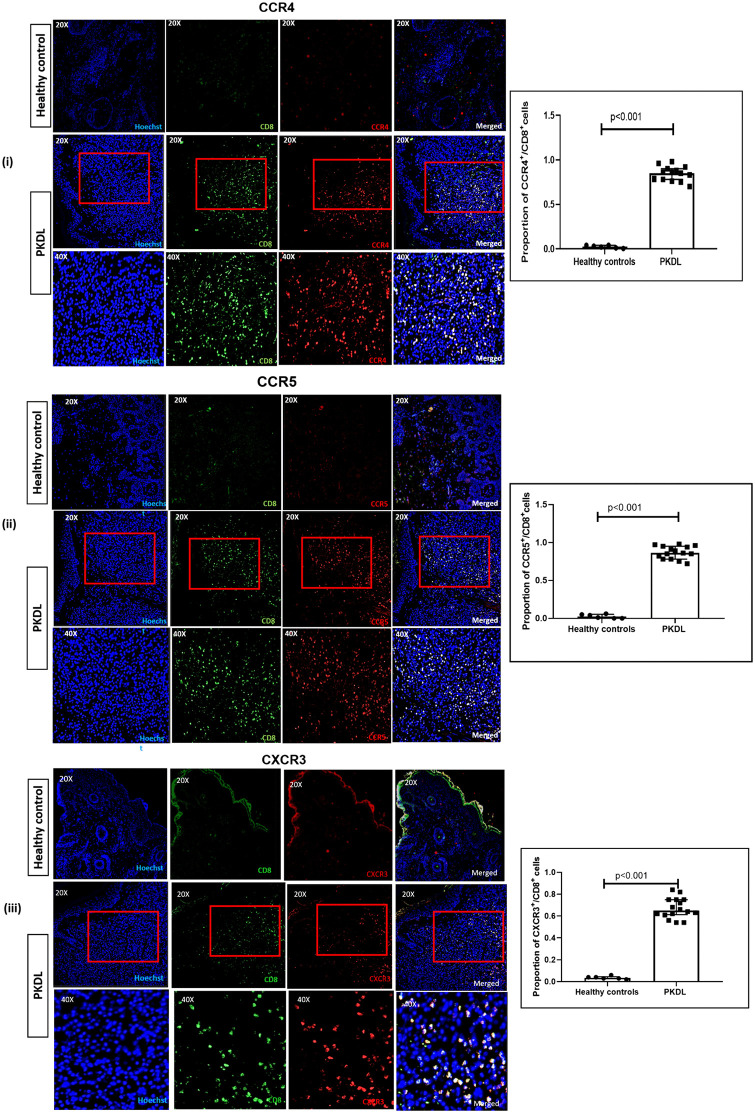
Elevated dermal expression of T cells associated chemokine receptors in PKDL. **i-iii:** Representative immunofluorescence profile showing the expression of chemokine receptors [red]; CCR4 **(i)**, CCR5 **(ii)** and CXCR3 **(iii)**, along with CD8 [green] positive cells in dermal biopsies of patients with PKDL (magnification, 20X, 40X). Co-localization of CD8^+^ with CCR4^+^, CCR5^+^ or CXCR3^+^ cells are indicated as ‘merged’ and counterstained with Hoechst [blue]. Bar scatter plots showing the proportion of double positive cells [CD8^+^/CCR4^+^, CD8^+^/CCR5^+^ and CD8^+^/CXCR3^+^] in healthy individuals (●) and patients with PKDL (■). Each horizontal bar represents the median (IQR).

The T cell chemokine receptors CCR4 (for CCL17/22), CCR5 (for CCL3/4/5) and CXCR3 (for CXCL9/10) were examined at lesional sites of PKDL patients. Patients with PKDL in comparison to healthy controls demonstrated a raised proportion of CD8^+^CCR4^+^, CD8^+^CCR5^+^ and CD8^+^CXCR3^+^ being [64.0(48.0-74.0) vs. [3.5(2.0–6.0) cells/field]; [53.0(48–74) vs. [4(2.75–5.75) cells/field] and [18.5(14.25-21.0) vs. [3.0(2.0–5.5) cells/field] respectively (**Fig 5**i-5iii).

### Increased T cells chemoattractants and their receptors in circulation of patients with PKDL

Given the enhanced dermal presence of T cell chemoattractants in PKDL (**Fig 4**), the circulating levels of chemokines, CCL3/4/5/17, CXCL9/10 and cytokines (IFN-ϒ, IL-5/8, TNF-α) was also determined. There was a significant upregulation in the circulating levels of T cell chemoattractants (**Table 3**), maximal enhancement being for CCL3, CCL17 and TNF-α. The number of CD8^+^ T cells correlated positively with CCL3/5/17, CXCL 9/10, IFN-ϒ and TNF-α (**Table 3**).

**Table 3:** Circulating levels of CD8^+^T cells chemo-attractants in PKDL.

Chemokines/ Cytokines	PKDL (pg/ml) [n=20]	Healthy controls (pg/ml) [n=9]	p value	Correlation with CD8 ^+^ cells/field
CCL3	30.95(7.35-78.28)	2.06(1.4-2.73)	p < 0.001	0.64, p < 0.05
CCL4	1095(485.8-2387)	427.4(372.1-555.1)	p < 0.05	0.49
CCL5	18347(7203-24344)	5978(4713-9696)	p < 0.05	0.53, p < 0.05
CCL17	65.67(41.24-108.3)	9.22(7.63-9.09)	p < 0.01	0.75, p < 0.05
CXCL9	2216.0(982.7-30766.0)	825.3(593.8-1557.0)	p < 0.05	0.65, p < 0.05
CXCL10	8087(2718-20926)	2229(1906-2985)	p < 0.01	0.56, p < 0.05
IFN-ϒ	108.9(53.1-221.3)	34.2(26.2-49.2)	p < 0.01	0.51, p < 0.05
IL-4	28.3(12.1-80.3)	11.8(8.78-23.43)	ns	0.13
TNF-α	81.1(43.7-131.9)	11.5(9.3-17.5)	p < 0.01	0.66, p < 0.05

The circulating levels of CD8^+^T cells chemoattractants were measured in patients with PKDL and healthy controls as described in Materials and methods. Values are stated in median (IQR).

**Fig 6:**
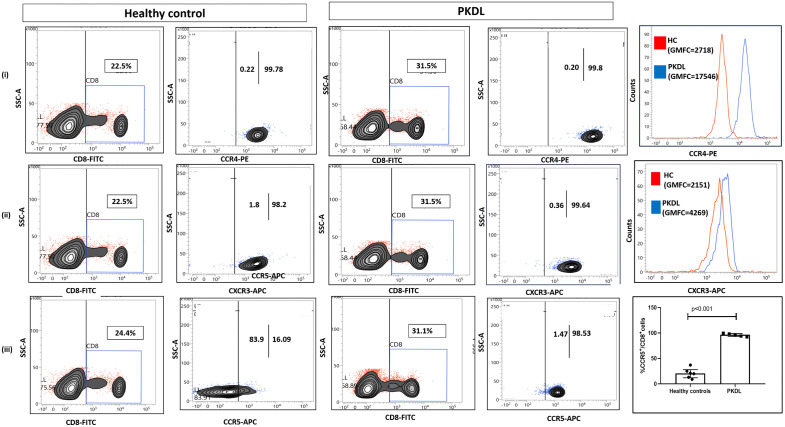
Circulatory status of chemokine receptors in CD8^+^ T cells in PKDL. **i-iii**: Representative contour plots of CD8^+^CCR4^+^ **(i),** CD8^+^CXCR3^+^ (**ii)** and CD8^+^CCR5^+^ **(iii)** T cells in peripheral blood of a healthy control and a patient with PKDL, along with histogram profiles of a healthy control (red line) and a patient with PKDL (blue line) along with bar scatter plot for frequency of CCR5^+^ cells within CD8^+^ T cells of healthy controls (●, n = 6) and at disease presentation (■, n = 5). Each horizontal bar represents the median (IQR).

The status of chemokine receptors CXCR3, CCR4/5 was determined in peripheral blood from healthy controls (n = 6) and patients with PKDL (n = 5) by flow cytometry. Lymphocytes from whole blood were initially morphologically gated based on FSC-A and SSC-A, and considered as ‘P1’; the CD8^+^ T cells were then identified within this population, and their status of CCR4^+^, CXCR3^+^, and CCR5^+^ was examined. In healthy controls vs. PKDL cases, the frequency of circulating CD8^+^/CCR4^+^ and CD8^+^/CXCR3^+^ cells was comparable, being [99.8(94.2-100.6) % vs. 99.7(96.23-102.45) %] and [99.6(96.2-104.2) % vs. 98.2(93.33-105.45) %] (Fig 6i, 6ii). However, the frequency of CD8^+^/ CCR5^+^ population in patients with PKDL was significantly higher than healthy controls, being [96.8(93.2-98.6) % vs. 20.5(12.0-28.7) %] (Fig 6iii). In terms of expression, the geometric mean fluorescence channel (GMFC) of CCR4^+^ and CXCR3^+^ in PKDL cases was 2.0 and 6.1-fold higher than healthy controls, being 4269 (4115–4874) vs. 2151 (2029–2547)]and [17161 (13282–18722) vs. 2817 (2150–3332)], respectively (**Fig 6**i, 6ii).

### Status of the pro-inflammatory milieu in patients with PKDL

**Fig 7:**
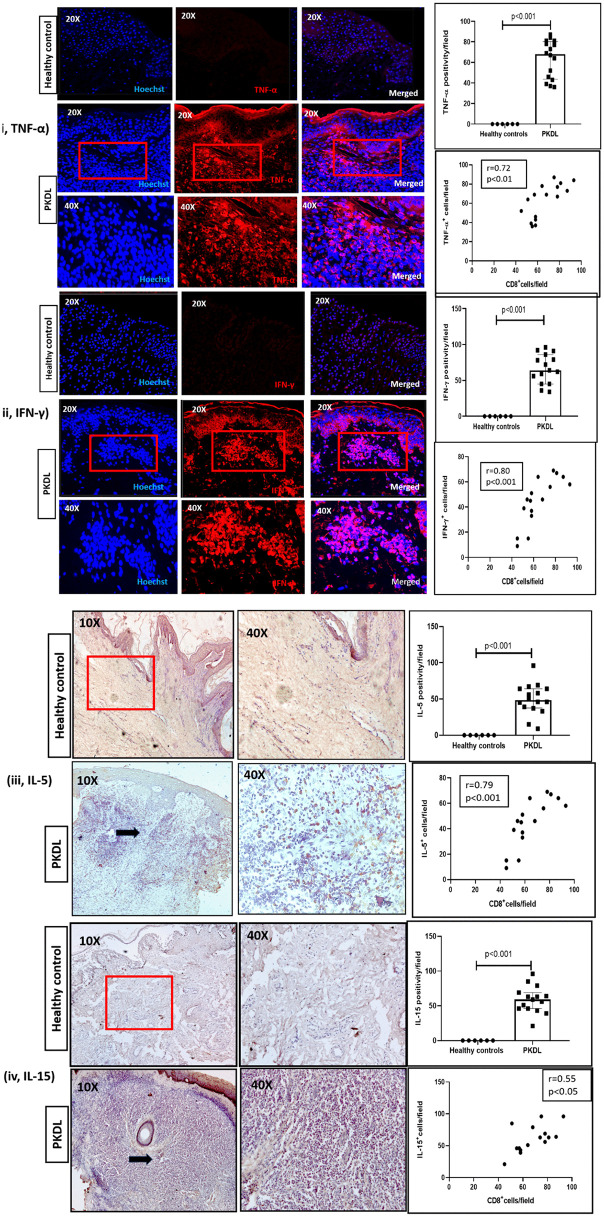
Dermal status of pro-inflammatory cytokines in PKDL. **i, ii:** Representative immunofluorescence profile in a healthy control and patient with PKDL showing the expression of TNF-α **(i)** and IFN-ϒ **(ii)** in dermal biopsies of a healthy control or patient with PKDL at 20 and 40X magnification along with bar scatter plots. Each horizontal bar represents the median (IQR) of healthy controls (●, n = 6) and patients with PKDL (■, n = 16). Correlation between the number of TNF-α^+^ **(i)** and IFN-ϒ^+^ cells **(ii)** with CD8^+^T cells at the lesional sites of patients with PKDL. **iii, iv**: Representative immunohistochemical profile of IL-5^+^ (**iii)** and IL-15^+^ **(iv)** cells from dermal biopsies of a healthy control and patient with PKDL (10x and 40x magnification) along with bar scatter plots indicating the IL-5^+^ (**iii)** and IL-15^+^ **(iv)** in healthy controls (●, n = 6) and patients with PKDL (■, n = 16). Each horizontal bar represents the median (IQR). Correlation of IL-5^+^ (**iii)** and IL-15^+^ **(iv)** positive cells (counts/field) with CD8^+^T cells (counts/field) in patients with PKDL.

Studies have demonstrated that TNF-α and IFN-γ drives CD8^+^ T cell migration by upregulating expression of CCR5 and CXCR3 respectively [18]. In PKDL lesions as compared to healthy individuals, an enhanced expression of TNF-α and IFN-γ was evident being [43.7(36–68)] and [64(45–86)] cells/field respectively, whereas it was absent in healthy controls (**Fig 7**i, 7ii). In view of the increased expression of CCR5 and CXCR3 (Fig 6), their plasma levels were examined and found to be significantly raised in PKDL cases, and positively correlated with the proportion of lesional CD8^+^ T cells (**Table 3**).

In the dermis of PKDL cases, the number of TNF-α^+^ and IFN- γ^+^ cells significantly correlated with infiltration of CD8^+^ T cells (r = 0.72 and r = 0.80 respectively, **Fig 7**i, 7ii). Given the role of IL-5 and IL-15 in driving migration of CD8^+^ T cells via upregulation of CCR4 [19] and CCR5 expression respectively [20], their expression was examined at lesional sites. During active disease, there was a strong expression of IL-5 [48(37–64)] and IL-15 [59(46–69)] cells/field, and was practically absent in healthy controls (**Fig 7**iii, 7iv).

### Functional role of lesional CD8^+^T cells in patients with PKDL

**Fig 8:**
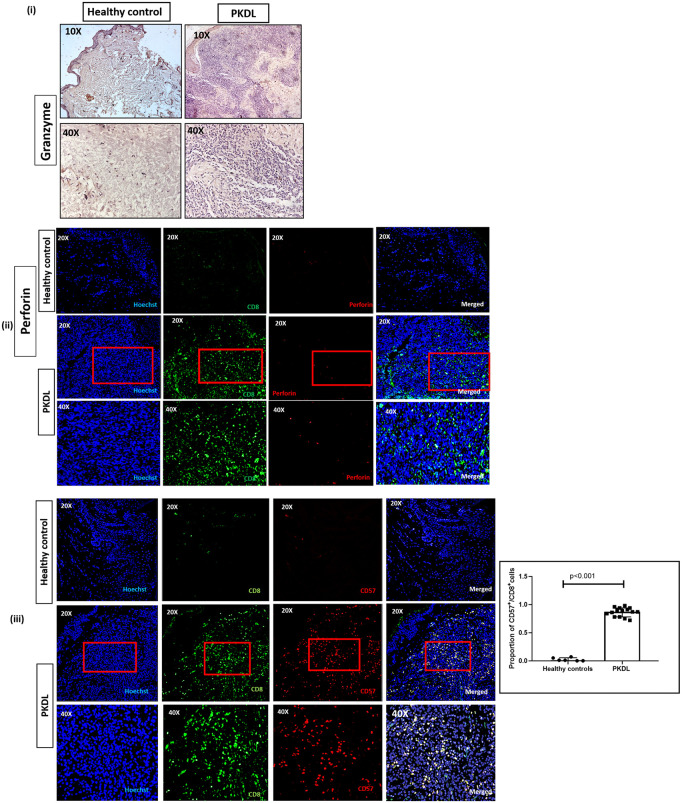
Impairment of the activation status of lesional CD8^+^ T cells in PKDL. **(i)**: Representative immunohistochemical profile of granzyme in dermal biopsies from a healthy control and patient with PKDL (10X and 40X magnification). **(ii, iii):** Representative immunofluorescence profile showing the expression of perforin [**ii**, red] and CD57 [**iii**, red], within CD8 [green] positive cells in dermal biopsies of a patient with PKDL (magnification, 20X and 40X). Co-localization of CD8 with perforin^+^ or CD57^+^ cells are indicated as ‘merged’ and counterstained with Hoechst [blue]. Bar scatter plot showing the proportion of CD8^+^/CD57^+^ cells **(iii)** in healthy individuals (●, n = 6) and patients with PKDL (■, n = 16). Each horizontal bar represents the median (IQR).

To assess the cytotoxic potential of lesional CD8^+^ T cells, the lesional status of perforin-1 and Granzyme-B was examined. Despite an overwhelming presence of CD8^+^ T cells, PKDL cases showed a conspicuous absence of Granzyme and Perforin **(**Fig 8i, 8ii), splenic tissue served as positive control (S1 Fig). As pro-inflammatory cytokines like IL-8 and TNF-α promote cellular senescence [21] and given the enhanced pro inflammatory milieu (Fig 7, Table 2), the expression of CD57 was evaluated. Patients with PKDL demonstrated a raised proportion of CD8^+^/CD57^+^ cells as compared to healthy controls, being [78.0(49.0-95.0) vs. 3.0(2.0–5.5) cells/field] (Fig 8iii**).**

## Discussion

Leishmaniases presents a broad spectrum of clinical manifestations affecting the skin and mucosal surfaces, each characterized by distinct patterns of tissue involvement and immunological responses. A unique histopathological hallmark across all forms of dermal leishmaniasis is the infiltration of immune cells. In cutaneous leishmaniasis (CL) caused by *L. major*, lesions typically display a dense and diffuse inflammatory dermal infiltrate composed predominantly of lymphocytes, macrophages, and plasma cells, accompanied by a localized granulomatous response that may contribute to effective parasite containment [22]. In contrast, PKDL caused by *L. donovani* exhibits epidermal alterations such as hyperkeratosis, papillomatosis, and epidermal atrophy [5], while mucocutaneous leishmaniasis (MCL) is marked by an inflammatory infiltrate enriched with neutrophils, followed by CD4⁺ and CD8 ⁺ T lymphocytes, plasma cells, and histiocytes, particularly surrounding necrotic and perinecrotic regions [23]. In comparison to healthy controls, the degree of cellular infiltration in PKDL was higher (Fig 1) as compared to healthy controls. Notably, this cellular infiltrate contained a high proportion of alternatively activated CD68 ⁺ macrophages, which likely function as parasitic reservoirs within the dermal infiltrate [24]. PKDL lesions displayed a striking predominance of CD8 ⁺ T cells (**Fig 2**), along with an absence of CD4 ⁺ T cells [16]. While CD4 ⁺ Th1 cells are indispensable for protective immunity, CD8 ⁺ T cells very likely contributed to parasite control and tissue damage through their cytotoxic features, as demonstrated in CL and MCL [25].

The recruitment of CD8 ⁺ T cells to sites of *Leishmania* infection is a critical determinant of disease outcome and is largely orchestrated by chemokine-receptor interactions. In CL caused by *L. braziliensis*, lesions display high expression of the CXCR3 ligands, CXCL9 and CXCL10 [26], consistent with findings that CXCR3 deficient mice exhibit impaired recruitment of CD4⁺ and CD8 ⁺ T cells to *L. major* infected lesions [9]. Similarly, in mouse models of *L. donovani* infection, CXCL10 is essential for attracting CD8 ⁺ T cells following vaccination, underscoring the importance of the CXCR3 axis in CD8 ⁺ T cell recruitment across the varied presentations of leishmaniasis [27]. In *L. braziliensis* infected skin, *CCR5* is the most highly expressed chemokine receptor transcript relative to healthy skin, and its ligands *CCL3 and CCL4* correlated with prolonged healing, suggesting a role for CCR5-mediated recruitment in sustaining disease [28]. Supporting this, CCR5 deficiency in CD8 ⁺ T cells resulted in their reduced accumulation and ameliorated pathology in murine *L. braziliensis* infection [29]. In PKDL caused by *L. donovani*, CD8 ⁺ T cells dominate the dermal infiltrate and displayed elevated expression of CCR4, along with its ligands CCL17 and CCL22, which declined post-treatment, implicating this axis in dermal homing [16]. As the disease progresses in *L. braziliensis* induced CL, CD8 ⁺ T cells become increasingly abundant within lesions, while CD4 ⁺ T cell frequency remains relatively stable [9]. Consistent with this, a model of controlled human infection using sandfly transmitted *L. major* demonstrated a high frequency of CD8 ⁺ T cells in lesions, particularly in recurrent infection or late-stage disease [30].

In PKDL cases, the Ki-67 negative status (**S2 Fig**) along with elevated levels of chemokines **(Table 3),** especially CXCL9/10, CCL3/4/5 facilitated the recruitment of T cells. Cytokine-mediated induction of chemokines is a key mechanism governing immune-cell trafficking and dermal leishmaniasis display distinct cytokine signatures that shape the lesion-specific immune microenvironment. For example, in self-healing CL, a mixed Th1/Th2 response dominated by Th1 cytokines IFN-γ, TNF-α, IL-12, IL-4, and IL-1 contributed to parasite control, but also induced collateral tissue damage [31]. In contrast, DCL lesions exhibited elevated IL-2, IL-4, and counter regulatory IL-10 with unchanged IFN-γ levels, reflecting an immunoregulatory environment that promotes disease chronicity [32]. In PKDL, the cytokine milieu is more complex, showing a simultaneous elevation of pro-inflammatory cytokines (TNF-α, IFN-γ, IL-1β, IL-6), the anti-inflammatory cytokine IL-4, and immunoregulatory mediators such as TGF-β and IL-10, collectively contributing to its chronic and persistent nature [15,33].

Cytokines can trigger the expression of chemokines, acting as crucial signaling mediators that amplify the immune response [34]. In PKDL, the most significantly enriched genes included cytokine/chemokine receptor binding and their activity, (Fig 3) and the prime upregulated chemokines included CCL3, CCL4, CCL5, CCL17, CCL22, CXCL9 and CXCL10 **(Fig 4).** Cytokines like TNF-α and IL-1 can directly trigger the release of multiple chemokines, such as CCL2, CCL5, and CXCL8 [34]. Treatment with IFN-γ can significantly increase the expression of chemokines like CXCL9, CXCL10, and CXCL11, which by binding to CXCR3 receptor can facilitate recruitment of activated T and NK cells [35]. IL-4 can trigger the production of specific chemokines like the CC chemokines CCL11 (eotaxin), CCL26, and CCL17 (TARC) [36]. This was endorsed in PKDL, evident by the increased presence of IL-5, TNF-α and IFN-γ (**Fig 7**). The enhanced frequency of CD8^+^CCR4^+^/CCR5^+^/CXCR3^+^ T cells in circulation and dermal lesions promoted the dermal extravasation of CD8^+^ T cells (Figs 2,5,6). However, these lesional CD8^+^ T cells lacked effector function molecules, e.g., granzyme and perforin as also showed features of cellular senescence (Fig 8). In view of granzyme and perforin remaining unchanged in circulating CD8^+^ T cells [16], it may be proposed that these anergic and exhausted CD8^+^ T cells did not migrate from circulation, and instead became exhausted following dermal homing [16]. Cellular senescence impacts not only on intracellular events, but also can potentially alter the immune environment [21,37–39]. Different pro inflammatory cytokines/chemokines are master regulators of the ‘senescence associated secretory phenotype (SASP)’ [40]. Cells undergoing cellular senescence can produce IL-1A, IL-1B, IL-6, IL-7, IL-8, IL-11, IL-15, IL-13, IL-10, CCL-27 and TNF-α [41], and a similar scenario was demonstrated in PKDL (Fig 7).

In tegumentary leishmaniasis, lesional transcriptomic analysis revealed a robust co-induction of senescence and pro-inflammatory gene signatures, suggesting the critical role of senescent T cells in orchestrating pathology [42]. Excessive T cell activation as observed during the early stages of CL is associated with their senescence and circulating senescent cells in infected individuals have increased potential to home to the skin as indicated by high expression of the skin homing receptor CLA (cutaneous lymphocyte antigen) [43]. The senescence-related secretion of inflammatory cytokines by these cells may facilitate control of infection and also contribute to the immunopathology of the disease [43]. Patients with Disseminated Leishmaniasis (DL) demonstrate an increased frequency of cytotoxic senescent CD8^+^ T cells as compared to patients with CL, and by promoting the lysis of infected cells, release of *Leishmania* to the extracellular compartment, contributed to the spread of parasites. [44]. T cells in a senescence state, generally express markers such as killer cell lectin-like receptor sub family G (KLRG-1) and CD57 [45,46]. In PKDL, the increased expression of CD57 coupled with an enhanced lesional expression of senescence-associated signature genes, *KLRG1*, *CDKN2A (*p  ≤ 0.05) and *NFKB2 (*p ≤ 0.05), the |log₂ fold change| ≥ 1.5 endorsing the senescent status of CD8^+^ T cells (**Fig 8**).

Taken together, in PKDL patients, the heightened Th1/Th2 cytokine environment drives the increased production of T cell attracting chemokines, possibly more within the skin than in circulation, thereby generating a chemotactic gradient, which then promoted the dermal homing of circulating CD8 ⁺ T cells. Upregulation of corresponding chemokine receptors on CD8 ⁺ T cells facilitated their migration into the dermis, where these cells within a pro-inflammatory environment acquired a senescent, functionally exhausted phenotype. Ideally, to prove that the senescence was associated with parasite persistence, performing functional assays would have allowed for better interpretation, but could not be implemented owing to the limited availability of dermal tissue. Together, these alterations in all likelihood account for the impairment of CD8^+^ driven cytotoxic responses and contribute to parasite persistence (S3 Fig). Parasite persistence in dermal lesions of patients with PKDL has important clinical and epidemiological implications, as these dermal parasites being easily accessible to the sandfly vector, can play a major role in disease transmission.

## Materials and methods

### Ethics statement

Archival material was used following approval from the Institutional Ethics committee of Institute of Postgraduate Medical Education & Research & Seth Sukhlal Karnani Memorial Hospital Hospital, and written informed consent was obtained from the individual or their legally accepted representative.

### Study population

The study population included biological material (plasma and formalin fixed paraffin embedded skin biopsies), sourced from patients diagnosed with PKDL (n = 24). The patients were enrolled 2018 onwards following passive surveillance at the Dermatology Outpatient Department, College of Medicine and Sagore Datta Hospital, Kolkata (n = 5, 2022-date), or active surveillance (n = 19, 2018-date) that was conducted in VL endemic districts of West Bengal (Malda, n = 11, and Dakshin Dinajpur, n = 3), Bihar (n = 2) and Jharkhand (n = 1, Sengupta et. al., 2019a); additionally, two cases were from non VL endemic districts of West Bengal (Burdwan, n = 1 and Nadia, n = 1). The cases were selected using standard case definitions (https://www.who.int/publications/i/item/9789241505215, last accessed on 6^th^ March, 2026), i.e., a history of VL, presenting with skin lesions (presence of papules, macules and/or nodules) and rK39 positivity. A dermal biopsy (4 mm) was collected at disease presentation from macules for the macular form (n = 7), and nodules for the polymorphic form (n = 9), avoiding whenever possible aesthetically sensitive areas (e.g., face); the cases were confirmed as PKDL by quantitative PCR [17]. As a control group, 17 healthy volunteers (preputial skin from six boys undergoing voluntary circumcision, skin biopsies from two healthy controls, along with peripheral blood from nine healthy controls). They were recruited from VL non-endemic areas and were seronegative for anti-leishmanial antibodies [15]. Studies have endorsed that the preputial skin histology is similar to adult skin [5,47]. Peripheral blood (5.0 ml) was collected in heparinized vials, and plasma stored at -80℃ for further use.

### Reagents

All reagents were obtained from Sigma Aldrich (St. Louis, MO, USA) except anti-human CD8 (clone C6/144B), IL-15 (Invitrogen, CA, USA), CCL3/4/5/22 (Invitrogen, CA, USA), CXCL9/10 (Proteintech, IL,USA), IL-5 (Proteintech, IL, USA), CCL17 (Proteintech, IL, USA), granzyme(Abcam, UK), anti-human perforin-Alexa Fluor647(AF-647), CD8-Fluorescein isothiocyanate (FITC), CCR4-Phycoerythrin (PE), CCR5-allophycocyanin(APC), CXCR3-allophycocyanin(APC), CD57 allophycocyanin(APC), TNF-α- Phycoerythrin (PE), IFN-γ- Phycoerythrin (PE) [BD Biosciences, San Jose, CA, USA], antifadent [Southern Biotech, Birmingham, AL, USA], EnVision FLEX Target Retrieval Solution [Dako, CA, USA] and secondary detection system [DAB + chromogen, ScyTek Laboratories, West Logan, UT, USA], Bio-Plex Pro Human Chemokine Panel 48-plex (for CXCL9/10, CCL3/4/5/17, IFN-γ, IL-4, TNF-α and IL-5, BioRad, CA, USA), Hoechst/trihydrochloride-trihydrate (Invitrogen, MA, USA), rK39 immunochromatographic strip test (InBios International, WA, USA), QIAmp DNA Mini kit (Qiagen, Hilden, Germany), SYBR Green qPCR Master Mix (Applied Biosystems, NY, USA).

### Histopathology

FFPE tissues were sectioned (3 μm), placed on poly L-lysine coated slides and examined by Hematoxylin and Eosin (H&E). Five fields were manually counted under a light microscope (Carl Zeiss, Germany) at 40X magnification and analyzed using Q path 0.4.2. software (version 0.4.2, Belfast, Ireland). Total cellular infiltration was assessed in polymorphic and macular PKDL by counting cells in five randomly selected or cell enriched fields respectively at 40X magnification, averaging the values, and expressing results as the total number of positive cells per field. In H&E staining, each hematoxylin-positive cell nucleus was considered as a ‘positive cell,’ while in IHC/IF, chromogen/fluorescence-positive cells with hematoxylin/Hoechst-counterstained nuclei were classified as ‘positive cell’.

### Immunohistochemistry

Sections (FFPE) were deparaffinised in xylene and rehydrated using descending grades of alcohol (100–70%) and distilled water. After heat induced epitope retrieval at pH 9, or at pH 6, the slides were incubated for 1 hr at room temperature (RT) with appropriate dilutions of primary antibody CD8 (1:25), CCL3 (1:20), CCL4 (1:20), CCL5 (1:20), CCL17 (1:100), CCL22 (1:100), CXCL9 (1:200), CXCL10 (1:200), IL-5 (1:100) and IL-15 (1:200).The slides were then washed with Tris-buffered saline containing 0.05% Tween-20 (0.02M, pH 7.4, TBS-T), incubated with anti-polyvalent HRP polymer at RT for 1 hr, and finally stained with DAB+ chromogen (1:100) at RT for 15 minutes as per the manufacturer’s protocol, and counter stained with haematoxylin. For healthy controls, 4 mm skin biopsies were obtained from foreskin of males who underwent voluntary circumcision. DAB-positive cells appeared brown and were analyzed using Q path 0.4.2. software. For the polymorphic form, the cell distribution was diffuse; accordingly, five fields were randomly selected, whereas for macular lesions, five ‘cell enriched’ fields were identified and cells counted under a light microscope (Carl Zeiss, Germany) at 40X magnification, averages taken and expressed as positive cells per field.

### Immunofluorescence

Sections of FFPE skin biopsies coated on Poly-L-lysine coated slides were deparaffinized in xylene followed by rehydration in graded alcohol. The slides were incubated with NH_4_Cl (5 mM) for 20 min. to reduce autofluorescence. For antigen retrieval, slides were placed in a pre-warmed antigen retrieval solution (citrate buffer, pH 6.0) and incubated for 20 minutes. The slides were then brought to RT (approximately 30 minutes) and washed with phosphate buffered saline (PBS, 0.02M). After blocking the non-specific binding sites with PBS + 3% bovine serum albumin + 5% fetal bovine serum for 45 minutes, they were incubated overnight at 4°C with fluorescent conjugated anti- human CCR4-PE, CXCR3-APC, CCR5-APC, CD57-APC, TNF-α-PE and IFN-γ-PE (diluted 1:100 in blocking buffer). Slides were washed thrice with PBS-Tween 20 (0.01% PBS-T) for 1 hr and a final wash with PBS for 10 min. Slides were counterstained with Hoechst (1 μg/mL, 50 μL, 10 min.), mounted with anti-fadent and stored at -20℃ for imaging. The images were captured in 20X using a Thunder imaging system (Leica, Wetzlar, Germany) and analyzed via Q path 0.4.2. software [14].

### Imaging analysis

Imaging for histopathology and immunohistochemistry-based assays was conducted using a light microscope (Carl Zeiss, Germany) at 10X and 40X magnification. Tissue quantification (H&E, IHC, IF) was performed using QuPath (Quantitative Pathology), an open-source image analysis software (version 0.4.2, Belfast, Ireland). In case of chemokines that were diffusely present in lesional sites, individual cell positivity was not attempted; instead, their expression was stated as positive or negative. Immunofluorescence of TNF-α and IFN-γ was assessed based on mean fluorescence intensity. The intensity was quantified as the amount of positive fluorescence per field at 40X magnification using QuPath. All analysis using the QuPath software were performed using the ‘merged’ FITC-PE/APC images, and a constant sigma factor as also threshold intensity cut-off was maintained. Similar brightness and contrast settings were applied to all images. All analyses with Qu Path were conducted on ‘merged’ images. Immunofluorescence imaging was performed using Thunder imaging systems (Leica Microsystems, WZ, Germany) at 20X magnification, respectively.

## Quantification of circulating cytokines and chemokines

Circulating levels of CCL3/4/5/17, CXCL9/10, IL-5/15, IFN-γ and TNF-α was measured in samples (diluted 1:4 with sample diluent), using a multiplex detection kit as per the manufacturer’s instructions. Data acquisition was carried out on a Luminex 200 Labmap system (Luminex, TX, USA) and analyzed using BioPlex Manager software version 6.2. The concentrations were determined by interpolation from a standard curve.

### Measurement of parasite load by quantitative PCR (qPCR)

To measure parasite load, a standard curve was generated by adding a defined number of *Leishmania* parasites (10–1 × 10^5^) from an *L. donovani* strain (MHOM/IN/83/AG83) to 180 μL of blood from a healthy control and real-time PCR was conducted as previously described [17]. Template DNA (0.1µg) was used, and the data expressed as parasites/µg of genomic DNA following extrapolation from a standard curve.

### Immunophenotyping

Whole blood (100 μL) was stained with anti-human FITC-conjugated CD8, as also CD8 cell surface receptors CCR4-Phycoerythrin (PE), CCR5-allophycocyanin (APC), CXCR3-allophycocyanin (APC). Following a 20-min incubation at room temperature (20–25°C), erythrocytes were lysed by incubating in BD Fix-lyse RBC lysing buffer for 10 min (2 mL) in dark at RT, washed twice with PBS and finally resuspended in PBS (400 μL) for acquisition in a flow cytometer (BD FACS Verse).

### Post Kala Azar Dermal Leishmaniasis (PKDL) Genome wide transcriptional profiling by RNA-seq

Total RNA was isolated from skin tissue sourced from patients with PKDL (n = 4) and unrelated healthy individuals (n = 3) using the Qiagen RNeasy Mini Kit (Hilden, Germany) according to the manufacturer’s protocol. RNA Quality was checked by using the TapeStation (Agilent) and samples with RNA Integrity Number, RIN > 7 was considered for RNA-sequencing. Library preparation for Genome-wide total RNA-sequencing was performed by using the KAPA RNA HyperPrep Kit with RiboErase (HMR) kit, followed by 100 bp paired-end sequencing using the Illumina NovaSeq 6000 platform to obtain raw sequencing reads followed by data analysis. To analyse the raw reads, steps included (a) data pre-processing: quality assessment of FASTQ files using FASTQC, followed by (b) adapter trimming and filtering out low-quality reads (threshold of MAPQ ≥ 30) using Cutadapt, (c) alignment to the human genome (GRCh37) using RNA-seq aligner STAR, followed by (d) normalization and (e) quantification of read counts. The raw counts for each annotated RNA transcript were obtained by using HTSeq-Count. Finally, the differentially expressed RNA transcripts were determined by using DESeq2. To consider differentially expressed RNA transcripts, the filtering criteria used was |log_2_ (fold change)| ≥ 1.5 and p-value ≤ 0.05. A functional pathway enrichment analysis was performed using ShinyGO (version 0.85.1), and an FDR cutoff ≤ 0.05 was used for selection of the top enriched pathways.

### Statistical analysis

Data was tested for normality, the data being non-parametric was analysed between groups using the Kruskal-Wallis’s test followed by Dunn’s multiple comparison test and between two groups, Mann-Whitney test was used; correlations were assessed using Spearman’s rank correlation, a correlation coefficient (r) ≥ ± 0.4 was considered relevant. Results are expressed as median with interquartile range (IQR). Statistical analyses were performed using GraphPad Prism (version 8.0.1, GraphPad Software Inc.), with p < 0.05 considered significant.

## Supporting information

RNA – sequencing data from dermal biopsies of patients with PKDL and healthy controls in this study are availabale at the NCBI GEO Database with reference number GSE324689.

S1 Fig Status of perforin and granzyme in splenic tissue. **i:** Representative immunohistochemical profile of splenic tissue showing the expression of granzyme (10X and 40X magnification). **ii:** Representative immunofluorescence profile of splenic tissue showing the expression of perforin [red] within CD8 [green] positive cells (magnification, 20X and 40X). Co-localization of CD8^+^ with perforin^+^ cells are indicated as ‘merged’ images.
(TIF)

S2 Fig Status of Ki67 in patients with PKDL. Representative immunohistochemical profile showing the expression of Ki67 in a breast carcinoma tissue, along with dermal biopsies from a healthy control and patient with PKDL (10X and 40X magnification).
(TIF)

S3 Fig CD8^+^ mediated cellular interactions in patients with PKDL. In patients with PKDL, an elevated Th1/Th2 cytokine milieu enhanced the expression of T cell chemoattractants, CCL3/4/5/17 and CXCL9/10, possibly more in the dermis than circulation, thereby generating a chemotactic gradient. These chemokines upregulated their corresponding chemokine receptors (CCR4/5, CXCR3) on circulating CD8 ⁺ T cells, resulting in an enhanced migration of CD8^+^ T cells from peripheral blood to skin. These recruited CD8 ⁺ T cells in the dermis exhibited features of senescence and functional exhaustion, evidenced by an increased expression of CD57 and reduced expression of cytotoxic molecules, perforin and granzyme, collectively generating an immune milieu that facilitated parasite survival and disease progression.
(TIF)

S1 Data The minimal data set includes all raw data utilized in the statistical analyses presented in the figures, ensuring transparency and reproducibility of the results. (XLSX)
