# Supplementary material for: Role of chemokines and their receptors in lesional CD8⁺ T cell homing in Indian Post-Kala-Azar Dermal Leishmaniasis
Source: PLoS Negl Trop Dis. 2026 Jul 24;20(7):e0014474. doi: 10.1371/journal.pntd.0014474 (PMC13399330; doi:10.1371/journal.pntd.0014474)
Supplement: Attachment — Submitted filename: response to reviewers comments.docx [file pntd.0014474.s006.docx]

**Title: Role of chemokines and their receptors in lesional CD8⁺ T cell homing in Indian Post-Kala-Azar Dermal Leishmaniasis**

# **Authors:** Shriya Saha^1^, Deep Goswami^1^, Mehelana Saha^1^, Bidhan Chakraborty^2^, Soham Saha^3^, Saikat Karuri^3^, Madhurima Roy^1^, Surya Jyati Choudhuri^4^, Nilay Kanti Das^5^, Uttara Chatterjee^6^, Raghunath Chatterjee^3^, Mitali Chatterjee^#^*.

**Manuscript Number:** PNTD-D-26-00553

| **Comments, Editor** | **Reply by the author(s)** | **Page and line number** | |
| --- | --- | --- | --- |
| **Reviewer 1** | | | |
| Authors could add information about the clinical stage of the disease, and if the patients received any treatment administration | The study population (n = 24) included a comparable proportion of patients with PKDL presenting with polymorphic (papular/nodular) or macular (hypopigmented) lesions. | Page 5, Lines 97-99 | |
|  | All cases were naïve, and had not received any  treatment at the time of collection of skin biopsies and/or blood. |  | |
| Discussion section needs improvement. Authors mention the figures numbers in almost all of the content of the discussion, leading to a repetition of the Results section. | Modified, reference to figures has been reduced |  | |
| Authors conclude that the senescent, exhausted phenotype together with the alteration of CD8+ T cytotoxic responses contribute to parasite persistence. This finding is helpful to advance in the topic but it could be accompanied by discussion of the clinical implications of the parasite persistence in the pathology together with the limitations of the analysis | Clinical implications:  Parasite persistence in dermal lesions of patients with PKDL has important clinical and epidemiological implications as these dermal parasites are easily accessible to the sandfly vector, and thereby play a major role in disease transmission. | Page 11,  295-297 | Lines |
|  | Limitations:  Ideally, to prove that senescence was associated with parasite persistence, performing functional assays | Page 11,  291-293 | lines |

|  | would have allowed for better interpretation. However, the limited availability of dermal tissue was a logistic limitation, |  | |
| --- | --- | --- | --- |
| Authors should verify the references referred to the use of preputial skin and its similarity to adult skin. | Studies using the preputial skin have reported that the histology is similar to adult skin [Sengupta et al., 2019b; Michalak-Micka et al., 2022], and can therefore be considered as representative of skin from a healthy individual. | Page 13,  326, 327. | Lines |
| **Reviewer 2** | | | |
| In Table 2, CCR5 is incorrectly listed as a receptor for IL-15. | Corrected | Page 19, Table 2 | |
| In Figure 3A (i–iii), there appear to be differences between groups. However, statistical significance is not indicated. Please clarify whether statistical analyses were performed and, if so, annotate accordingly. This issue applies to additional  figures throughout the manuscript. | Corrected and modified | Figures 5, 7, 8. | |
| Senescent CD8 T cells are not clearly defined. The reliance on CD57 expression alone is insufficient, and the associated limitations are not discussed.  Also, please provide a more comprehensive definition of T cell senescence. | Senescent T cells are defined by markers, (i) killer cell lectin-like receptor sub family G (KLRG-1), (ii) CD57 [46, 47] (iii) reduction in co-stimulatory molecules (CD27 and CD28). | Page 11,  282-285, | Lines |
|  | In PKDL, as compared to healthy individuals, transcriptomic analysis revealed an enhanced lesional expression of senescence-associated signature genes, *KLRG1*, *CDKN2A (*p ≤ 0.05) and  *NFKB2 (*p ≤ 0.05), the \|log₂ fold change\| ≥ 1.5. | Figure 8 |  |
| Please provide a reference for the statement in lines 505–506. | Incorporated | Page 11, Line 277  Reference no-44 | |
| Figure legend titles and Results section subheadings are overly general and do not | Modified | Line 455 | |
| adequately convey the key findings. Please provide more descriptive headings. |  | Line 463 | |
|  |  | Line 471,472 | |
|  |  | Line 480, 486 | |

|  |  | 494  Line 501  Line 513, 523 |
| --- | --- | --- |
| In Figure 2A (iii), gene labels are difficult to read and should be larger. | Modified | Figure 3 |
| Line 406: Please clarify what is meant by “perforin-H.” | Corrected as perforin-1 | Page 8, Line 199 |
| The Results section is overly dense and includes extensive statistical detail that would  be more appropriately placed in figure legends, which detracts from readability. | In Results section, the p values have been omitted  from the text and incorporated into the figures. | Incorporated into  figures |

| **Journal Requirements (Additional changes to be made to the manuscript)** | | |
| --- | --- | --- |
| what are the novel findings in this current manuscript in relation to your previous  publication (Scientific Reports volume 9, Article number: 762 (2019); Mukherjee et al). | **Key advances in the present study include:** |  |
|  | Our earlier work focussed on establishing the presence of anergic CD8⁺ T cells in dermal lesions of PKDL. In the current study, we have extended this observation by demonstrating the enhanced presence of T cell chemoattractants (CCL3, CCL4, CCL5, CCL17, CXCL9, CXCL10), along with an increased expression of their corresponding receptors (CCR4, CCR5, CXCR3), and they mediate the dermal homing of CD8⁺ T cells. |  |
|  | The present work aimed to provide a comprehensive overview regarding the status of T cell chemokines and their receptors, in circulation and dermal lesions using multiple approaches, e.g. bioplex assay, flow cytometry, transcriptomics and  immunohistochemistry/ immunofluorescence. |  |
| We do not publish any copyright or trademark symbols that usually accompany proprietary names, eg ©, ®, or TM (e.g. next to drug or reagent names). Therefore please remove all instances of trademark/copyright symbols throughout the text, including:  - TM on page: 6. | Corrected | Page 13,  Line 341 |
| Your manuscript's sections are not in the correct order. Please amend to the following order:  Abstract, Introduction, Results, Discussion, and Methods | Modified |  |
| Please upload all main figures as separate Figure files in .tif or .eps format. For more | Modified and uploaded as per NAAS |  |
| information about how to convert and format your figure files please see our guidelines: | guidelines. |  |
| <https://journals.plos.org/plosntds/s/figures> |  |  |

| We notice that your supplementary Figures are included in the manuscript file. Please remove them and upload them with the file type 'Supporting Information'. Please ensure that each Supporting Information file has a legend listed in the manuscript after the references  list. | Corrected | Page 29, Stated as supporting information  Lines 659-  675 |
| --- | --- | --- |
| We note that your Data Availability Statement is currently as follows: "The data that support the findings of this study are included in the manuscript.". Please confirm at this time whether or not your submission contains all raw data required to replicate the results of your study. Authors must share the “minimal data set” for their submission. PLOS defines the minimal data set to consist of the data required to replicate all study findings reported in the article, as well as related metadata and methods (<https://journals.plos.org/plosone/s/data-availability#loc-minimal-data-set-definition>).  For example, authors should submit the following data:   1) The values behind the means, standard deviations and other measures reported; 2) The values used to build graphs; 3) The points extracted from images for analysis..   Authors do not need to submit their entire data set if only a portion of the data was used in the reported study.  If your submission does not contain these data, please either upload them as Supporting Information files or deposit them to a stable, public repository and provide us with the relevant URLs, DOIs, or accession numbers. For a list of recommended repositories, please see <https://journals.plos.org/plosone/s/recommended-repositories>.  If there are ethical or legal restrictions on sharing a de-identified data set, please explain them in detail (e.g., data contain potentially sensitive information, data are owned by a third-party organization, etc.) and who has imposed them (e.g., an ethics committee). Please also provide contact information for a data access committee, ethics committee, or other institutional body to which data requests may be sent. If data are owned by a third party,  please indicate how others may request data access. | Uploaded as minimal dataset file |  |
| Please provide a detailed Financial Disclosure statement. This is published with the article. It must therefore be completed in full sentences and contain the exact wording you wish to be published.1) Please clarify all sources of financial support for your study. List the grants, grant numbers, and organizations that funded your study, including funding received from your institution. Please note that suppliers of material support, including research materials, should be recognized in the Acknowledgements section rather than in the Financial  Disclosure2) State the initials, alongside each funding source, of each author to receive each grant. For example: "This work was supported by the National Institutes of Health (#######to AM; ###### to CJ) and the National Science Foundation (###### to AM)."3) State what role the funders took in the study. If the funders had no role in your study, please state: "The funders had no role in study design, data collection and analysis, decision to publish, or preparation of the manuscript."4) If any authors received a salary from any of your funders, please state which authors and which funders..If you did not receive any funding for this study, please simply state: u201cThe authors received no specific funding for this work.u201d. However, your funding information on the submission form indicates receiving fund from Anusandhan National Research Foundation.  Please indicate by return email the full and correct funding information for your study and confirm the order in which funding contributions should appear. Please be sure to indicate whether the funders played any role in the study design, data collection and analysis,  decision to publish, or preparation of the manuscript. | Modified | Pages 11, 12,  Lines 300-308 |
| Some material included in your submission may be copyrighted. According to PLOS's copyright policy, authors who use figures or other material (e.g., graphics, clipart, maps) from another author or copyright holder must demonstrate or obtain permission to publish this material under the Creative Commons Attribution 4.0 International (CC BY 4.0) License used by PLOS journals. Please closely review the details of PLOS's copyright requirements here: PLOS Licenses and Copyright. If you need to request permissions from a copyright holder, you may use the PLOS Content Copyright Permission Form Please respond directly to this email and provide any known details concerning your material's license terms and permissions required for reuse, even if you have not yet obtained copyright permissions or are unsure of your material's copyright compatibility. Once you have responded and addressed all other outstanding technical requirements, you may resubmit your manuscript within Editorial Manager. Potential Copyright Issues:  i) 2i, and Graphical abstract.: Please confirm whether you drew the images / clip-art within the figure panels by hand. If you did not draw the images, please provide (a) a link to the source of the images or icons and their license / terms of use; or (b) written permission from the copyright holder to publish the images or icons under our CC BY 4.0 license. Alternatively, you may replace the images with open source alternatives. See these open source resources you may use to replace images / clip-art: - [https://commons.wikimedia.org](https://commons.wikimedia.org/)  - <https://openclipart.org/> | The cliparts are free and available online. |  |

**Response to additional comments:**

| **Comment** | **Response** | **Page no: Line no:** |
| --- | --- | --- |
| We note that the author byline has changed since the last version of the manuscript. PLOS follows the Committee on Publication Ethics (COPE: https://publicationethics.org) guidelines for changes to authorship and therefore requires specific information relating to these changes. We would be grateful if you could, therefore, please complete and return an Authorship Change Form, which may be downloaded from the following link  https://journals.plos.org/plosntds/s/authorship#loc-authorship-changes  Once you have provided a completed form, the change request will be evaluated by PLOS staff for approval. | Change of authorship form uploaded |  |
| The following file is currently uploaded as file type 'Other', which is not viewable by the reviewers: Minimal DATA SET.xlsx. Please change the file type to 'Supporting Information' and include a legend in the manuscript if you wish it to be included in review | Modified and uploaded as supporting information. |  |
| We have amended your Competing Interest statement to comply with journal style. We kindly ask that you double check the statement and let us know if anything is incorrect. | Checked and verified as correct. |  |
| Please ensure that the affiliations of the author (Bidhan Chakraborty) listed on the manuscript title page (CITY) do exactly match with the affiliations provided in the online submission form  NOTE: Affiliations should include a department (if applicable), an institution, a CITY, and a country | Verified |  |
| Thank you for stating "Archival material was used following approval from the Institutional Ethics committee of Institute of Postgraduate Medical Education & Research & SSKM Hospital, and written informed consent was obtained from the individual or their legally accepted representative."Please state the full name of SSKM | The full name of SSKM has been stated as Seth Sukhlal Karnani Memorial Hospital. | Page 13  Line 331 |
| The file inventory includes files for Figures 1A,1B, 2A,2B,3A,3B. We would recommend either combining these into a single Figure file with separate internal panels, or renumbering them as individual figures, as we are not able to publish multiple components of a single figure as separate files.  NOTE: Please ensure that all included figures are referenced to ensure that your paper is fully reviewed. | Figure 1A modified to Figure 1,  Figure 1B modified to Figure 2, Figure 2A modified to Figure 3, Figure 2B modified to Figure 4, Figure 3A modified to Figure 5, Figure 3B modified to Figure 6, Figure 4 modified to Figure 7, Figure 5 modified to Figure 8, Figure 6 modified to Figure 9. Accordingly, result section, discussion and figure legends have been corrected. | Page 5  Lines 114, 115, 117, 122, 123  Page 6  Lines 125, 130, 133, 134, 136, 139, 140, 153, 154.  Page 7  Lines 163, 166, 178, 180, 182, 183  Page 8  Lines 189, 190, 194, 197, 200, 202, 204, 206.  Page 9  Lines 219, 221.  Page 10  Line 257, 258, 263, 265, 266, 273.  Page 11  Line 287,297.  Page 21  Line 457, 465, 473, 481, 486, 494, 501, 513, 523. |
| Please provide a detailed Financial Disclosure statement. This is published with the article. It must therefore be completed in full sentences and contain the exact wording you wish to be published. 1) Please clarify all sources of financial support for your study. List the grants, grant numbers, and organizations that funded your study, including funding received from your institution. Please note that suppliers of material support, including research materials, should be recognized in the Acknowledgements section rather than in the Financial Disclosure2) State the initials, alongside each funding source, of each author to receive each grant. For example: "This work was supported by the National Institutes of Health (####### to AM; ###### to CJ) and the National Science Foundation (###### to AM)."3) State what role the funders took in the study. If the funders had no role in your study, please state: "The funders had no role in study design, data collection and analysis, decision to publish, or preparation of the manuscript."4) If any authors received a salary from any of your funders, please state which authors and which funders. If you did not receive any funding for this study, please simply state: u201cThe authors received no specific funding for this work.u201d | This work received financial assistance awarded to SS from a National Post doctoral fellowship from ANRF, Govt. of India (PDF/2023/000349). The study received technical support from a Dioraphte Foundation grant (CfP-NTD2023) and JC Bose grant, ANRF, Govt. of India ANRF/JBG/2025/000445/HAA awarded to MC, Multidisciplinary Research Unit (MRU) at IPGME&R, Kolkata, India [Multidisciplinary Research Unit (MRU), Department of Health Research (DHR), Govt. of India, V.25011/611/2016-HR]. DG, MR, MS are recipients of a Senior Research Fellowship from Dept of Biotechnology, University Grants Commission, and Council for Scientific and Industrial Research, Govt. of India respectively. The funders had no role in study design, data collection and analysis, decision to publish, or preparation of the manuscript. We are thankful to the Department of Health and Family Welfare, West Bengal, for providing support for conducting the field surveys. | Page 11, 12,  Lines 300-309 |
