# Supplementary material for: Role of chemokines and their receptors in lesional CD8⁺ T cell homing in Indian Post-Kala-Azar Dermal Leishmaniasis
Source: PLoS Negl Trop Dis. 2026 Jul 24;20(7):e0014474. doi: 10.1371/journal.pntd.0014474 (PMC13399330; doi:10.1371/journal.pntd.0014474)
Supplement: Peer review history

# Peer review: Decision Letter 0

**DOI:** 10.1371/journal.pntd.0014474.r001

**Authors:** Susan Madison-Antenucci

28 Apr 2026

-->PNTD-D-26-00553

Role of chemokines and their receptors in lesional CD8⁺ T cell homing in Indian Post-Kala-Azar Dermal Leishmaniasis

PLOS Neglected Tropical Diseases

Dear Dr. Chatterjee,

Thank you for submitting your manuscript to PLOS Neglected Tropical Diseases. After careful consideration, we feel that it has merit but does not fully meet PLOS Neglected Tropical Diseases's publication criteria as it currently stands. Therefore, we invite you to submit a revised version of the manuscript that addresses the points raised during the review process.

Please submit your revised manuscript within by Jun 27 2026 11:59PM. If you will need more time than this to complete your revisions, please reply to this message or contact the journal office at plosntds@plos.org. When you're ready to submit your revision, log on to https://www.editorialmanager.com/pntd/ and select the 'Submissions Needing Revision' folder to locate your manuscript file.

Please include the following items when submitting your revised manuscript:

* A letter that responds to each point raised by the editor and reviewer(s). You should upload this letter as a separate file labeled 'Response to Reviewers'. This file does not need to include responses to any formatting updates and technical items listed in the 'Journal Requirements' section below.

* A marked-up copy of your manuscript that highlights changes made to the original version. You should upload this as a separate file labeled 'Revised Manuscript with Track Changes'.

* An unmarked version of your revised paper without tracked changes. You should upload this as a separate file labeled 'Manuscript'.

If you would like to make changes to your financial disclosure, competing interests statement, or data availability statement, please make these updates within the submission form at the time of resubmission. Guidelines for resubmitting your figure files are available below the reviewer comments at the end of this letter.

We look forward to receiving your revised manuscript.

Kind regards,

Camila I. de Oliveira

Academic Editor

PLOS Neglected Tropical Diseases

Susan Madison-Antenucci

Section Editor

PLOS Neglected Tropical Diseases

Shaden Kamhawi

co-Editor-in-Chief

PLOS Neglected Tropical Diseases

orcid.org/0000-0003-4304-636XX

Paul Brindley

co-Editor-in-Chief

PLOS Neglected Tropical Diseases

orcid.org/0000-0003-1765-0002

**Additional Editor Comments :**

Dear Dr. Chatterjee,

Two experts in the field have evaluated your manuscript. While the two referees have stated the work presented is sound and an advancement to the field, they both raised concerns that preclude publication in its current format. I therefore ask that you carefully read the comments and suggestions and submit a revised version of the manuscript. In this revised version, please highlight all modifications made. I kindly ask that you clarify in your rebuttal and also include this clarification in the revised version: what are the novel findings in this current manuscript in relation to your previous publication (Scientific Reports volume 9, Article number: 762 (2019); Mukherjee et al). Please also submit a point-by-point response to both reviewers' comments, paying particular attention to those from Referee #1 (the suggestions for improvement of the text are embedded within the general appreciation of the work, sections Methods, Results, and Conclusions).

We look forward to receiving a modified version.

Regards

PLoS NTDs

**Journal Requirements:**

If the reviewer comments include a recommendation to cite specific previously published works, please review and evaluate these publications to determine whether they are relevant and should be cited. There is no requirement to cite these works unless the editor has indicated otherwise.

1) We do not publish any copyright or trademark symbols that usually accompany proprietary names, eg ©,  ®, or TM  (e.g. next to drug or reagent names). Therefore please remove all instances of trademark/copyright symbols throughout the text, including:

- TM on page: 6.

2) Your manuscript's sections are not in the correct order.  Please amend to the following order: Abstract, Introduction, Results, Discussion, and Methods

3) Please upload all main figures as separate Figure files in .tif or .eps format. For more information about how to convert and format your figure files please see our guidelines:

https://journals.plos.org/plosntds/s/figures

4) We notice that your supplementary Figures are included in the manuscript file. Please remove them and upload them with the file type 'Supporting Information'. Please ensure that each Supporting Information file has a legend listed in the manuscript after the references list.

5) We note that your Data Availability Statement is currently as follows: "The data that support the findings of this study are included in themanuscript.". Please confirm at this time whether or not your submission contains all raw data required to replicate the results of your study. Authors must share the “minimal data set” for their submission. PLOS defines the minimal data set to consist of the data required to replicate all study findings reported in the article, as well as related metadata and methods (https://journals.plos.org/plosone/s/data-availability#loc-minimal-data-set-definition).

For example, authors should submit the following data:

1) The values behind the means, standard deviations and other measures reported;

2) The values used to build graphs;

3) The points extracted from images for analysis..

Authors do not need to submit their entire data set if only a portion of the data was used in the reported study.

If your submission does not contain these data, please either upload them as Supporting Information files or deposit them to a stable, public repository and provide us with the relevant URLs, DOIs, or accession numbers. For a list of recommended repositories, please see https://journals.plos.org/plosone/s/recommended-repositories.

If there are ethical or legal restrictions on sharing a de-identified data set, please explain them in detail (e.g., data contain potentially sensitive information, data are owned by a third-party organization, etc.) and who has imposed them (e.g., an ethics committee). Please also provide contact information for a data access committee, ethics committee, or other institutional body to which data requests may be sent. If data are owned by a third party, please indicate how others may request data access.

6) Please provide a detailed Financial Disclosure statement. This is published with the article. It must therefore be completed in full sentences and contain the exact wording you wish to be published.

1) Please clarify all sources of financial support for your study. List the grants, grant numbers, and organizations that funded your study, including funding received from your institution. Please note that suppliers of material support, including research materials, should be recognized in the Acknowledgements section rather than in the Financial Disclosure

2) State the initials, alongside each funding source, of each author to receive each grant. For example: "This work was supported by the National Institutes of Health (####### to AM; ###### to CJ) and the National Science Foundation (###### to AM)."

3) State what role the funders took in the study. If the funders had no role in your study, please state: "The funders had no role in study design, data collection and analysis, decision to publish, or preparation of the manuscript."

4) If any authors received a salary from any of your funders, please state which authors and which funders..

If you did not receive any funding for this study, please simply state: u201cThe authors received no specific funding for this work.u201d

7) Your current Financial Disclosure states, "The author(s) received no specific funding for this work.".

However, your funding information on the submission form indicates receiving fund from Anusandhan National Reseaarch Foundation.

Please indicate by return email the full and correct funding information for your study and confirm the order in which funding contributions should appear. Please be sure to indicate whether the funders played any role in the study design, data collection and analysis, decision to publish, or preparation of the manuscript.

8) ) Some material included in your submission may be copyrighted. According to PLOS's copyright policy, authors who use figures or other material (e.g., graphics, clipart, maps) from another author or copyright holder must demonstrate or obtain permission to publish this material under the Creative Commons Attribution 4.0 International (CC BY 4.0) License used by PLOS journals. Please closely review the details of PLOS's copyright requirements here: PLOS Licenses and Copyright. If you need to request permissions from a copyright holder, you may use the PLOS Content Copyright Permission Form Please respond directly to this email and provide any known details concerning your material's license terms and permissions required for reuse, even if you have not yet obtained copyright permissions or are unsure of your material's copyright compatibility. Once you have responded and addressed all other outstanding technical requirements, you may resubmit your manuscript within Editorial Manager. Potential Copyright Issues:

i) 2i, and Graphical abstract.: Please confirm whether you drew the images / clip-art within the figure panels by hand. If you did not draw the images, please provide (a) a link to the source of the images or icons and their license / terms of use; or (b) written permission from the copyright holder to publish the images or icons under our CC BY 4.0 license. Alternatively, you may replace the images with open source alternatives. See these open source resources you may use to replace images / clip-art: - https://commons.wikimedia.org - https://openclipart.org/

**Reviewers' Comments:**

Reviewer's Responses to Questions

**Key Review Criteria Required for Acceptance?**

As you describe the new analyses required for acceptance, please consider the following:

**Methods**

-Are the objectives of the study clearly articulated with a clear testable hypothesis stated?

-Is the study design appropriate to address the stated objectives?

-Is the population clearly described and appropriate for the hypothesis being tested?

-Is the sample size sufficient to ensure adequate power to address the hypothesis being tested?

-Were correct statistical analysis used to support conclusions?

-Are there concerns about ethical or regulatory requirements being met?

Reviewer #1: The objectives of the study are clearly articulated with a clear testable hypothesis stated. The study is focused in the expression of chemokines and chemokine receptors in dermal lesions from patients with PKDL. The study design is appropriate to address the stated objectives. The study includes the use of dermal biopsies and plasma samples from indian patients with PKDL and healthy subjects. Authors could add information about the clinical stage of the disease, and if the patients received any treatment administration. The sample is sufficient to ensure adequate power. The studied samples are considered very valuable as they correspond to skin biopsies, which are not often easy to obtain. Authors should verify the references referred to the use of preputial skin and its similarity to adult skin. The statistical analysis used are correct to support the conclusions. There are not ethical concerns, as authors declare that archival material was used "following approval from the Institutional Ethics committee of Institute of Postgraduate Medical Education & Research & SSKM Hospital, and written informed consent was obtained from the individual or their legally accepted representative". In the Methods section, authors should add some missing determinations as CD57, perforin and granzyme analysis.

Reviewer #2: (No Response)

**********

**Results**

-Does the analysis presented match the analysis plan?

-Are the results clearly and completely presented?

-Are the figures (Tables, Images) of sufficient quality for clarity?

Reviewer #1: The analysis presented match the analysis plan. There are several subtitles in the Results section, which are well designed. The section begins with the description of the clinical characteristics of the study population, followed by the analysis of the dermal cellular infiltrate, the predominance of CD8+ T cell infiltration in the lesions, the over expression of genes encoded for T cell homing receptors and ligands, the status of lesional T cell chemoattractants and their receptors. Then the study describes the findings related to peripheral blood samples, in relation to the circulating levels of chemoattractants and expression of their receptors. The results are clearly described. Figures from immunofluorescence and immunohistochemistry studies have the needed quality. Authors should verify if the journal allows separate figures 1A, 1B, 2A, 2B, etc. Authors should verify some of the figures, to present them in a uniform manner, in relation to showing or not statistical differences; letter size; percentages of expression / median fluorescence results, etc. Tables are clear and present enough data.

Reviewer #2: (No Response)

**********

**Conclusions**

-Are the conclusions supported by the data presented?

-Are the limitations of analysis clearly described?

-Do the authors discuss how these data can be helpful to advance our understanding of the topic under study?

-Is public health relevance addressed?

Reviewer #1: From my point of view, the Discussion section needs improvement. Authors mention the figures numbers in almost all of the content of the discussion, leading to a repetition of the Results section. Authors conclude that the senescent, exhausted phenotype together with the alteration of CD8+ T cytotoxic responses contribute to parasite persistence. This finding is helpful to advance in the topic but it could be accompanied by discussion of the clinical implications of the parasite persistence in the pathology together with the limitations of the analysis.

Reviewer #2: (No Response)

**********

**Editorial and Data Presentation Modifications?**

Use this section for editorial suggestions as well as relatively minor modifications of existing data that would enhance clarity. If the only modifications needed are minor and/or editorial, you may wish to recommend “Minor Revision” or “Accept”.

Reviewer #1: Within the following sections, I explain in more detail the suggested modifications in order to improve the manuscript.

Reviewer #2: (No Response)

**********

**Summary and General Comments**

Use this section to provide overall comments, discuss strengths/weaknesses of the study, novelty, significance, general execution and scholarship. You may also include additional comments for the author, including concerns about dual publication, research ethics, or publication ethics. If requesting major revision, please articulate the new experiments that are needed.

Reviewer #1: The present study is well designed and uses adequate methodologies to analyze valuable biological samples from patients with PKDL. Besides, the wide contribution of the research group for the advance of the pathology understanding is reflected in numerous high impact publications in the area. The present study focuses in different aspects of chemoattractant molecules, chemokine receptors, inflammatory genes, senescent and cytotoxic properties of CD8+ T cells in dermal lesions. As the manuscript present diverse and interesting findings, I would suggest to verify and explain the differences between this study and the publication of the group dated in 2019 (Sci. Rep, S. Mukherjee) entitled: "Impaired activation of lesional CD8+ T-cells is associated with enhanced expression of Programmed Death-1 in Indian Post Kala-azar Dermal Leishmaniasis". In that publication, authors remarked that dermal lesions from PKDL patients showed absence of perforin and granzyme, analyzing two time points: presentation of the disease and after treatment. Authors also assessed circulating levels of CCL17 and CCL22. These determinations were also assessed in the present work, giving high importance to the absence of cytotoxic granules. I suggest to review possible similarities between both publications, to avoid result repetition, and emphasize novel aspects of the present work.

Reviewer #2: This manuscript characterizes the immune environment of lesions from patients who develop post-kala-azar dermal leishmaniasis (PKDL), a major public health problem in many parts of the world. The authors use RNA sequencing, imaging analyses, and flow cytometry of both lesion-derived and circulating cells from patients and healthy controls to investigate mechanisms underlying the recruitment of CD8 T cells to the skin and their phenotypic features within lesions. This study is timely, as CD8 T cells play critical roles in both protection and pathology across a range of skin diseases. While substantial work has addressed these processes in cutaneous leishmaniasis, comparatively little is known about PKDL. Given the absence of a mouse model for this disease manifestation, studies in human patients are particularly valuable, and this group has contributed important advances to the field. The Introduction and Discussion are clear and well-written. However, the Results section is overly dense and includes extensive statistical detail that would be more appropriately placed in figure legends, which detracts from readability. Specific comments to improve clarity and presentation are outlined below.

Specific comments:

1. In Table 2, CCR5 is incorrectly listed as a receptor for IL-15.

2. In Figure 3A (i–iii), there appear to be differences between groups. However, statistical significance is not indicated. Please clarify whether statistical analyses were performed and, if so, annotate accordingly. This issue applies to additional figures throughout the manuscript.

3. Senescent CD8 T cells are not clearly defined. The reliance on CD57 expression alone is insufficient, and the associated limitations are not discussed. Also, please provide a more comprehensive definition of T cell senescence.

4. Please provide a reference for the statement in lines 505–506.

5. Figure legend titles and Results section subheadings are overly general and do not adequately convey the key findings. Please provide more descriptive headings.

6. In Figure 2A (iii), gene labels are difficult to read and should be larger.

7. Line 406: Please clarify what is meant by “perforin-H.”

**********

PLOS authors have the option to publish the peer review history of their article (what does this mean?). If published, this will include your full peer review and any attached files.

If you choose “no”, your identity will remain anonymous but your review may still be made public.

**Do you want your identity to be public for this peer review?**  For information about this choice, including consent withdrawal, please see our Privacy Policy.

Reviewer #1: No

Reviewer #2: No

**Figure resubmission:**

While revising your submission, we strongly recommend that you use PLOS’s NAAS tool (https://ngplosjournals.pagemajik.ai/artanalysis) to test your figure files. NAAS can convert your figure files to the TIFF file type and meet basic requirements (such as print size, resolution), or provide you with a report on issues that do not meet our requirements and that NAAS cannot fix.

-->

After uploading your figures to PLOS’s NAAS tool - https://ngplosjournals.pagemajik.ai/artanalysis, NAAS will process the files provided and display the results in the "Uploaded Files" section of the page as the processing is complete. If the uploaded figures meet our requirements (or NAAS is able to fix the files to meet our requirements), the figure will be marked as "fixed" above. If NAAS is unable to fix the files, a red "failed" label will appear above. When NAAS has confirmed that the figure files meet our requirements, please download the file via the download option, and include these NAAS processed figure files when submitting your revised manuscript.-->

**Reproducibility:**

To enhance the reproducibility of your results, we recommend that authors of applicable studies deposit laboratory protocols in protocols.io, where a protocol can be assigned its own identifier (DOI) such that it can be cited independently in the future. Additionally, PLOS ONE offers an option to publish peer-reviewed clinical study protocols. Read more information on sharing protocols at https://plos.org/protocols?utm_medium=editorial-email&utm_source=authorletters&utm_campaign=protocols

-->

# Author comment: Author response to Decision Letter 1

**DOI:** 10.1371/journal.pntd.0014474.r002

26 May 2026

Attachment Submitted filename: response to reviewers comments.docx

# Peer review: Decision Letter 1

**DOI:** 10.1371/journal.pntd.0014474.r003

**Authors:** Susan Madison-Antenucci

16 Jun 2026

Dear Dr. Chatterjee,

We are pleased to inform you that your manuscript 'Role of chemokines and their receptors in lesional CD8⁺ T cell homing in Indian Post-Kala-Azar Dermal Leishmaniasis' has been provisionally accepted for publication in PLOS Neglected Tropical Diseases.

Before your manuscript can be formally accepted you will need to complete some formatting changes, which you will receive in a follow up email. A member of our team will be in touch with a set of requests.

Please note that your manuscript will not be scheduled for publication until you have made the required changes, so a swift response is appreciated.

IMPORTANT: The editorial review process is now complete. PLOS will only permit corrections to spelling, formatting or significant scientific errors from this point onwards. Requests for major changes, or any which affect the scientific understanding of your work, will cause delays to the publication date of your manuscript.

Should you, your institution's press office or the journal office choose to press release your paper, you will automatically be opted out of early publication. We ask that you notify us now if you or your institution is planning to press release the article. All press must be co-ordinated with PLOS.

Thank you again for supporting Open Access publishing; we are looking forward to publishing your work in PLOS Neglected Tropical Diseases.

Best regards,

Camila I. de Oliveira

Academic Editor

PLOS Neglected Tropical Diseases

Susan Madison-Antenucci

Section Editor

PLOS Neglected Tropical Diseases

Shaden Kamhawi

co-Editor-in-Chief

PLOS Neglected Tropical Diseases

orcid.org/0000-0003-4304-636XX

Paul Brindley

co-Editor-in-Chief

PLOS Neglected Tropical Diseases

orcid.org/0000-0003-1765-0002

***********************************************************

p.p1 {margin: 0.0px 0.0px 0.0px 0.0px; line-height: 16.0px; font: 14.0px Arial; color: #323333; -webkit-text-stroke: #323333}span.s1 {font-kerning: none

Reviewer's Responses to Questions

**Key Review Criteria Required for Acceptance?**

As you describe the new analyses required for acceptance, please consider the following:

**Methods**

-Are the objectives of the study clearly articulated with a clear testable hypothesis stated?

-Is the study design appropriate to address the stated objectives?

-Is the population clearly described and appropriate for the hypothesis being tested?

-Is the sample size sufficient to ensure adequate power to address the hypothesis being tested?

-Were correct statistical analysis used to support conclusions?

-Are there concerns about ethical or regulatory requirements being met?

Reviewer #1: The objectives of the study are clearly articulated with a clear testable hypothesis. The study is well designed, with the use of multiple and adequate laboratory techniques. The population is appropriate and clearly described. The sample size is sufficient, highliting the value of the type of sample (skin biopsies) used in this work. The statistical analysis is correct. There are not ethical or regulatory requirements concerns.

Reviewer #2: (No Response)

**********

**Results**

-Does the analysis presented match the analysis plan?

-Are the results clearly and completely presented?

-Are the figures (Tables, Images) of sufficient quality for clarity?

Reviewer #1: The analysis presented match the analysis plan. Results are clearly and completely presented. I would suggest to remove data of median (range) from Results section, as authors showed these data within figures. Figures present sufficient quality for clarity.

Reviewer #2: (No Response)

**********

**Conclusions**

-Are the conclusions supported by the data presented?

-Are the limitations of analysis clearly described?

-Do the authors discuss how these data can be helpful to advance our understanding of the topic under study?

-Is public health relevance addressed?

Reviewer #1: The conclusions supported the data presented. The revised version of the manuscript has been improved, and authors took into consideration the suggestions of the reviewers. The limitations of the analysis are now clearly described. In the Discussion section, authors added lines about the importance of the findings for advancing the understanding of the topic under study, taking into account the relevance for public health.

Reviewer #2: (No Response)

**********

**Editorial and Data Presentation Modifications?**

Use this section for editorial suggestions as well as relatively minor modifications of existing data that would enhance clarity. If the only modifications needed are minor and/or editorial, you may wish to recommend “Minor Revision” or “Accept”.

Reviewer #1: My suggestions would be to avoid the use of median (range) along Results sections, when data is also shown inside figures. Besides, I would suggest to review some little grammatical errors along the manuscript. In the case of Figure 4, for example, authors could mention figure as follows (Fig. 4 i-vii), instead of naming the figure 7 times.

Reviewer #2: (No Response)

**********

**Summary and General Comments**

Use this section to provide overall comments, discuss strengths/weaknesses of the study, novelty, significance, general execution and scholarship. You may also include additional comments for the author, including concerns about dual publication, research ethics, or publication ethics. If requesting major revision, please articulate the new experiments that are needed.

Reviewer #1: From my point of view, this work gives interesting findings to advance in the knowledge of the field, using valuable skin samples from an adequate population of patients suffering Post-Kala-Azar Dermal Leishmaniasis. The work is well designed with the use of multiple techniques in order to validate their results. I believe that the manuscript has been improved from the original version. The revised version is readable with a complete an well written discussion.

Reviewer #2: The authors have appropriately addressed previous concerns.

**********

PLOS authors have the option to publish the peer review history of their article (what does this mean?). If published, this will include your full peer review and any attached files.

If you choose “no”, your identity will remain anonymous but your review may still be made public.

**Do you want your identity to be public for this peer review?**  For information about this choice, including consent withdrawal, please see our Privacy Policy.

Reviewer #1: No

Reviewer #2: No

# Editor report: Acceptance letter

**DOI:** 10.1371/journal.pntd.0014474.r004

**Authors:** Susan Madison-Antenucci

Dear Dr. Chatterjee,

We are delighted to inform you that your manuscript, "Role of chemokines and their receptors in lesional CD8⁺ T cell homing in Indian Post-Kala-Azar Dermal Leishmaniasis," has been formally accepted for publication in PLOS Neglected Tropical Diseases.

We have now passed your article onto the PLOS Production Department who will complete the rest of the publication process. All authors will receive a confirmation email upon publication.

The corresponding author will soon be receiving a typeset proof for review, to ensure errors have not been introduced during production. Please review the PDF proof of your manuscript carefully, as this is the last chance to correct any scientific or type-setting errors. Please note that major changes, or those which affect the scientific understanding of the work, will likely cause delays to the publication date of your manuscript. Note: Proofs for Front Matter articles (Editorial, Viewpoint, Symposium, Review, etc...) are generated on a different schedule and may not be made available as quickly.

Soon after your final files are uploaded, the early version of your manuscript will be published online unless you opted out of this process. The date of the early version will be your article's publication date. The final article will be published to the same URL, and all versions of the paper will be accessible to readers.

For Research Articles, you will receive an invoice from PLOS for your publication fee after your manuscript has reached the completed accept phase. If you receive an email requesting payment before acceptance or for any other service, this may be a phishing scheme. Learn how to identify phishing emails and protect your accounts at https://explore.plos.org/phishing.

Thank you again for supporting open-access publishing; we are looking forward to publishing your work in PLOS Neglected Tropical Diseases.

Best regards,

Shaden Kamhawi

co-Editor-in-Chief

PLOS Neglected Tropical Diseases

Paul Brindley

co-Editor-in-Chief

PLOS Neglected Tropical Diseases
